# Changes in Gel Structure and Chemical Interactions of *Hypophthalmichthys molitrix* Surimi Gels: Effect of Setting Process and Different Starch Addition

**DOI:** 10.3390/foods11010009

**Published:** 2021-12-21

**Authors:** Xin Jiang, Qing Chen, Naiyong Xiao, Yufan Du, Qian Feng, Wenzheng Shi

**Affiliations:** 1 College of Food Sciences & Technology, Shanghai Ocean University, Shanghai 201306, China; 13053523375@163.com (X.J.); talina_chen@126.com (Q.C.); Xny931215@163.com (N.X.); yfdu122433@163.com (Y.D.); 18361206059@163.com (Q.F.); 2 National Research and Development Center for Processing Technology of Freshwater Aquatic Products (Shanghai), Shanghai 201306, China

**Keywords:** surimi, starch, setting, water migration, microstructure, chemical interactions

## Abstract

The modifications of histological properties and chemical forces on heated surimi gels with starch addition (0–12 g/100 g surimi) were investigated. Two types of heating processes (direct heating and two-step heating) were carried out on surimi gels in order to reveal the effect of setting on mixed matrices. The results of transverse relaxation time showed less immobile water and free water converted into bound water in a matrix subjected to the setting process. Scanning electron microscope and light microscopy images revealed inefficient starch-swelling in two-step heated gels. Chemical interactions and forces in direct cooking gels were more vulnerable to starch addition, resulting in significant decreases in hydrophobic interaction and sulfhydryl content (*p* < 0.05). With the increment of starch, the disulfide stretching vibrations of the gauche–gauche–gauche conformation were reduced in both gel matrices. The structural variations of different components collectively resulted in changes in texture profile analysis and water holding capacity. Overall, the results demonstrated that starch addition had a great and positive effect on the weak gel matrix by direct heating.

## 1. Introduction

Surimi-based products have become an increasingly consumed food with prominent characteristics of convenience, special flavor, and unique texture [1]. In addition, the deep processing technology gave it the qualities of excellent nutritional value and high digestibility [2]. Due to the continuous overexploitation of marine fishing resources, low-value or cultured fish has been paid more attention as a potential alternative raw material for surimi production [3]. Silver carp (*Hypophthalmichthys molitrix*), widely cultured in China, is considered a low-commercial value fish due to its muddy flavor and higher by-product content [4]. However, due to its rapid growth, high yield, and low price, silver carp could replace sea fish as raw material for surimi [5]. Through the processes of rinsing, dehydration, and defatting, the myofibrillar protein could be well retained, and more fishy compounds were removed, which improved the flavor characteristics to a certain extent. Raw surimi could be further processed into prepared foods or ready-to-eat foods in order to satisfy different taste demands for surimi-based products, which realize the value addition of silver carp [6].

Throughout the entire process of thermal gel formation, heat treatment is an important step in determining the quality of the gel matrix [7]. Before heating, free myosin molecules and actomyosin complexes were dispersed, while the network structure complex reinforced by actomyosin was completed as the temperature rose [8]. Based on the unique property of fish protein, heating processes can be divided into direct heating and two-step heating, with significant differences in protein networks between these two treatments [9]. Direct heating treatment provided the gel with soft structure but might cause the formation of protein aggregates in different sizes [10]. In case of two-step heating, an extension of setting time at 4–40 °C prior to heating at 90 °C could strengthen gel properties, which was widely used in making kamaboko [6]. The first setting step, in which more available cross-linking sites become accessible, ensured a gradual sol-gel transition so that an orderly initial protein network formed [11,12]. Subsequent secondary heating at high temperature is used for the final production of thermal gels [13]. Compared with direct heated gels, the two-step heated gels enabled the gel structure to remain more compact even when suffering from physical forces [10]. Hence, the setting process played a vital role in gel processing. However, the addition of some exogenous substances during the actual processing was also necessarily for helping improve gel texture.

Starch, as a popular food additive for improving gels, plays an essential role in reducing the factory processing costs of surimi-based products and meeting consumer taste needs [14,15]. According to the “packing effect” raised by Kong et al. [16], the starch granules swelled and then exerted more pressure on the gel matrix, forming a firm and cohesive gel. Starch swelling in the surimi matrix increased the hardness of the gels, thereby improving overall gel properties. It also found that starch did not crosslink with the surimi protein, whereas it could change the chemical interaction in the surimi matrix [17]. Thus far, studies have mainly emphasized the effects of different starches in improving gel strength [17,18,19]. Nevertheless, the surimi gel network also affected the filling effect of starch in the mixture, which was rarely reported in research. It was hypothesized that the characteristics of the starch-containing surimi matrix could be affected by the setting processing, showing different change trends in various properties. Accordingly, two heating processes (direct heating process and two-step heating process) were set up to detect the different properties of the mixed matrix and to compare the systematic effect of the setting process on the heated starch–surimi matrix.

Therefore, the research aimed to elucidate water migration, microstructure, chemical interactions, and surimi protein structure in the starch–surimi matrix comprehensively. Physical properties, such as texture profile analysis, whiteness, and water holding capacity, were also detected, providing insight into the combined effect of thermal processing and exogenous additives on texture characteristics.

## 2. Materials and Methods

### 2.1. Materials and Reagents

Silver carp frozen surimi (AAA grade, cryoprotectants were 6% sucrose and 0.25% polyphosphate) obtained from Jinli Fishery Food Co., Ltd. (Honghu, China) was cut into pieces weighing about 200 g and stored at −20 °C after vacuum sealing. The moisture and crude protein contents were 75.20% and 14.37%, respectively. Native potato starch was purchased from Hangzhou Starpro Starch Co., Ltd. (Hangzhou, China). The P0006C Detergent Compatible Bradford Protein Assay Kit was obtained from Shanghai Beyotime Biotechnology Co., Ltd. (Shanghai, China). The other chemicals were analytical grade, with the exception of KBr (spectrography), and purchased from Sinopharm Chemical Reagent Co., Ltd. (Shanghai, China).

### 2.2. Sample Preparation

Frozen surimi was thawed at 4 °C overnight and cut into small pieces. An amount of 200 g of semi-thawed silver carp surimi was blended for 2 min and then mixed with 5 g of NaCl for 4 min. During the blending process, moisture content was adjusted to 80% with iced water that was also used to keep the temperature of mixtures below 10 °C. Native potato starch (0 g, 3 g, 6 g, 9 g, or 12 g/100 g surimi) was added to the surimi and mixed in blender (AM-CG108-1, Appliance Co. of America, Zhuhai, China) for 6 min. Then, starch–surimi combinations were filled into the plastic tubes with a diameter of 25 mm and heated in two different processes. In the direct heating process, the tubes were heated in a water bath at 90 °C for 30 min, obtaining cooking gel (CG). In the two-step heating process, a combination of preheating at 40 °C for 1 h and cooking at 90 °C for 30 min was carried out to obtain setting-cooking gel (SCG) [6]. After heating, all samples were stored at 4 °C for 12 h.

### 2.3. Low Field Nuclear Magnetic Resonance (LF-NMR)

The relaxation time and moisture distribution were measured by a Niumag Pulsed NMR analyzer (MesoMR23-060H-I, Niumag Electric Co., Shanghai, China). Gel samples were cut into the cylinders with a height of 20 mm, and a CPMG (Carr-Purcell-Meiboom-Gill) pulse sequence was carried out [20,21]. The CPMG parameters are listed in Table 1.

### 2.4. Scanning Electron Microscope (SEM)

The surimi gels were cut into 3 mm × 3 mm × 1.5 mm pieces and fixed with glutaraldehyde (2.5%, *v*/*v*) for 14 h at 4 °C. The fixed samples were rinsed with 0.1 M phosphoric acid buffer (pH 7.2–7.4) three times. After that, the samples were dehydrated with a serious of ethanol solution (30%, 50%, 70%, 80%, 90%, and 100%) and then replaced with tert-butanol solution (absolute ethanol: tert-butanol = 3:1, 1:1, 1:3, 0:1). The dehydrated samples were dried by using a freeze dryer (SCIENTZ-10N, Ningbo Scientz Biotechnology Co., Ltd., Ningbo, China) and sputter-coated with gold. The microstructures were analyzed by an SEM instrument (Hitachi SU5000, Hitachi High-Tech Co., Ltd., Shanghai, China) at an acceleration voltage of 5 kV.

### 2.5. Light Microscopy (LM)

Surimi gels were cut into 5 mm cubes and immersed with 10% formalin fixture solution (1:10, *w*/*v*) for more than 24 h. After being dehydrated with gradient alcohol (75%, 85%, 90%, 95%, and 100%), the samples were placed in xylene followed by wax leaching using an embedding machine (JB-P5, Wuhan Junjie Electronics Co., Ltd., Wuhan, China). After cooling at −20 °C, the paraffin slice was cut with a thickness of 4 μm carried out by a tissue spreader (KD-P, Zhejiang Kehua Instrument Co., Ltd., Jinhua, China). The dewaxed slices were stained using the periodic acid-Schiff (PAS) method according to the method of Jia et al. [22]. The samples were observed using a light microscope (MS500W, Shanghai Meizs Precision Instrument Co., Ltd., Shanghai, China).

### 2.6. Determination of Non-Covalent Bonds

The chemical interactions or bonds were determined according to the method of Yan et al. [23] with slight modifications. Briefly, 1 g of surimi gels was added 10 mL 0.05 M NaCl (A), 0.6 M NaCl (B), 0.6 M NaCl + 1.5 M urea (C), and 0.6 M NaCl + 8 M urea (D) and homogenized for 2 min by homogenizer (Shanghai Fokker Equipment Co. Ltd., Shanghai, China). The mixtures were placed at 4 °C for 1 h and subsequently centrifuged at 15,000× *g* for 10 min by refrigerated centrifuge (CR21GШ, Hitachi High-Tech Co., Ltd., Shanghai, China).

The protein concentrations of supernatant collected were determined by using the Biuret method [24]. The contents of nonspecific associations (A), ionic bonds (B), hydrogen bonds (C), and hydrophobic interactions (D) were determined by the concentrations of soluble protein in supernatants.

### 2.7. Determination of Total Sulfhydryl Groups

The measurement of total SH content was described by Zhang et al. [25]. According to the following equation, the content of SH was calculated.

(1) $$\mathrm{SH}\;\mathrm{groups}\;\mu \mathrm{mol}/\;g\;\mathrm{protein}=\frac{A\;\times \;D}{\varepsilon \;\times \;d\;\times \;c}\;\times \;10^{6}$$

In the equation, A and D represent absorbance at 412 nm and the dilution ratio, respectively; ε refers to molar extinction coefficient, 13,600 L/(mol·cm); d is the thickness of cuvette, 1 cm; and c is the protein concentration, 4 mg/mL.

### 2.8. Fourier Transform Infrared (FT-IR) Spectroscopy

The surimi gels were freeze-dried and ground into powder. An amount of 1.5 mg of powder was mixed with 150 mg KBr and then pressed into a transparent sheet. The spectrums of the matrix were observed by a Nicolet iS5 FT-IR spectrometer (Thermo Fisher Instruments Co., Ltd., Shanghai, China). Samples were then scanned 32 times from 400 to 4000 cm^−1^ with a resolution of 4 cm^−1^ [26]. Relative intensity was analyzed by OMNIC 9.2 software (Thermo Fisher Scientific Inc., Waltham, MA, USA) and PeakFit 4.12 software (Systat Software Inc., San Jose, CA, USA).

### 2.9. Texture Profile Analysis (TPA)

Equilibrium temperature to 25 °C, the gel matrix was cut into cylinders (20 mm × 20 mm), and TPA was determined by using a TA-XT Plus texture analyzer (Stable Micro Systems Ltd., Godalming, UK) with P/50 cylindrical probe. The pre-test speed and test speed were both 1 mm·s^−1^, the compression percentage was 40%, and the trigger force was 5 g.

### 2.10. Determination of Whiteness

The surimi gels were cut with a thickness of 10 mm. The L* (lightness), a* (redness/greenness), and b* (yellowness/blueness) values of gels were determined by colorimeter (CR-400, Konica Minolta Japan, Inc., Tokyo, Japan). Whiteness was calculated as follows.

(2) $$\begin{array}{c} {\mathrm{Whiteness}=100\;\text{−}\;[(100\;\text{−}\;L}^{*}{)}^{2}{+a}^{*}{}^{2}{+b}^{*}{}^{2}{]}^{1/2} \end{array}$$

### 2.11. Determination of Water Holding Capacity (WHC)

All samples were cut into 3 g with a thickness of about 5 mm, weighed (W_1_) and then placed into three layers of filter paper. After centrifugation of 15 min (5000× *g*, 4 °C), the gels were quickly weighed again (W_2_). The equation of water holding capacity is defined as follows:

(3) $$\begin{array}{c} \mathrm{Centrifugal}\;\mathrm{loss}\;\left(\%\right)\;=\;\frac{W_{2}}{W_{1}}\;\times \;100 \end{array}$$

(4) $$\mathrm{Water}\;\mathrm{holding}\;\mathrm{capacity}\;(\%)=\frac{M-\mathrm{CL}}{M}\times 100$$

where CL is the centrifugal loss of surimi gel after heating, and M is the moisture content determined by drying the sample at 105 °C until constant weight.

### 2.12. Statistical Analysis

All experiments were performed at least three times, and the results were presented as the mean ± standard deviation (SD). Statistical Package for Social Science (SPSS 26, SPSS Inc., Chicago, IL, USA) was used to carry out all statistical analysis with the significance level set at 0.05 based on one-way analyses of variance (ANOVA). Significant differences were evaluated by Duncan’s multiple range test (*p* < 0.05).

## 3. Results and Discussion

### 3.1. Changes of Relaxation Time and Water Distribution of Starch–Surimi Gels with Non-Setting or Setting Effect

**Figure 1:**
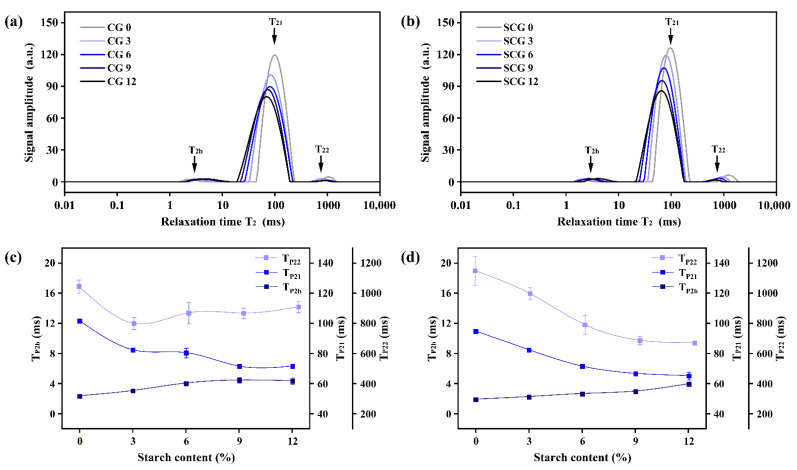
T_2_ relaxation time (**a**,**b**) and water mobility (**c**,**d**) of starch–surimi matrix in direct heating process and two-step heating process. CG: gel obtained by direct heating; SCG: gel obtained by two-step heating. 0: without starch; 3, 6, 9, and 12: incorporation with 3%, 6%, 9%, and 12% starch.

The results of LF-NMR can effectively determine water mobility and distribution in gel matrix [27]. T_2b_ (1–20 ms), T_21_ (20–300 ms), and T_22_ (400–2000 ms) represented the transverse relaxation time of bound water, immobilized water, and free water (Figure 1a, b), respectively. It was obvious the relaxation time of different water migrated. As shown in Figure 1c, d, the peak relaxation time of bound water (T_P2b_) in gels without starch was 2.39 ms (CG) and 1.94 ms (SCG), between which there was a significant difference (*p* < 0.05) that might be related to the gel structure heated by dissimilar processes. T_P2b_ increased by starch addition and showed a positive correlation with starch content. The migration of T_P2b_ was relevant to different water mobility bound by the protein and starch. The peak relaxation time of immobile water (T_P21_) dropped from 101.71 ms to 71.71 ms in CG, and from 94.84 ms to 65.36 ms in SCG. The shorter the relaxation time (T_2_), the stronger the binding force of water molecules to matrix [12]. Thus, the water mobility of bound water significantly increased with starch incorporation (*p* < 0.05), while the mobility of non-mobile and free water decreased in both matrices. This was consistent with the investigation by Li et al. [28], who found that starch showed a better restriction capacity on the free motion of water molecules. However, the relaxation time between the two types of gels had a significant difference (*p* < 0.05), indicating that starch-swelling differed in gel matrices with different heating treatments.

**Figure 2:**
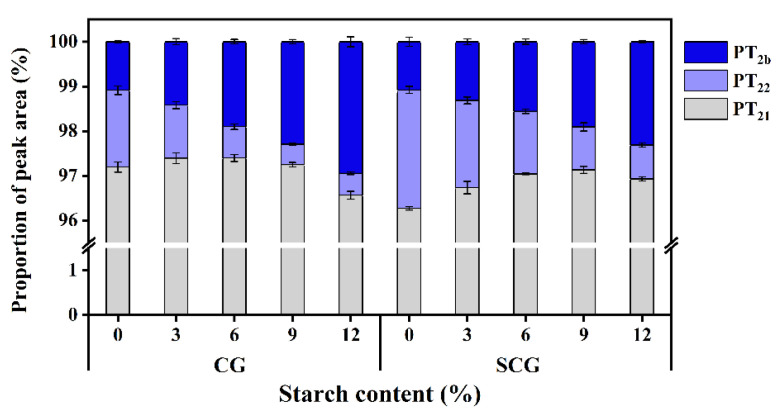
Peak area proportion (P_T2b_, P_T21_, and P_T22_) of different water in starch–surimi matrix subjected to direct heating and two-step heating. Caption: see Figure 1.

The relative moisture content was observed by peak area proportions (P_T2b_, P_T21_, and P_T22_) in Figure 2. It was discovered that immobile water made up the majority of the primary proportion in surimi gel, followed by bound water and free water. P_T21_ showed the relative content of immobile water that increased initially with the addition of starch, resulting in a reduction in free water in both CG and SCG. The P_T21_ of CG and SCG reached a maximum at 6% and 9% starch content, respectively. Subsequently, it decreased and was possibly associated with the dehydration of surimi gels suffered by the compression from starch swelling. Moreover, starch incorporation reduced the relative surimi content in the entire matrix, causing a decrease in immobile water content held by surimi gel network. Notably, in the non-starch containing gels, P_T21_ of CG and SCG was 97.20% and 96.27%, showing that the gel matrix had higher P_T21_ without preincubation at 40 °C. The changes might presumably contribute to overheated time and the squeeze by gel formation in SCG, resulting in a transition from immobile water to free water. Although direct heating formed a poor gel, the juiciness mouthfeel of surimi products improved [29]. Since the hydroxyl groups in starch bound more water, the matrix with starch incorporation significantly differed in P_T22_ (*p* < 0.05). However, P_T2b_ significantly increased with the continuous addition of starch (*p* < 0.05), especially in CG. With 12% starch, P_T2b_ of CG increased by 173.15% compared to 113.89% in SCG. Among the above changes, it indicated that water absorbed by starch existed in the form of immobile water and bound water. Moreover, it was inferred that the starch in direct cooking gels showed better swelling states, which resulted in the phenomena where the internal starch structure tended to easily combine with water.

### 3.2. Changes in Tissue Histology of Starch–Surimi Gels with Non-Setting or Setting Effect

#### 3.2.1. Light Micrograph

**Figure 3:**
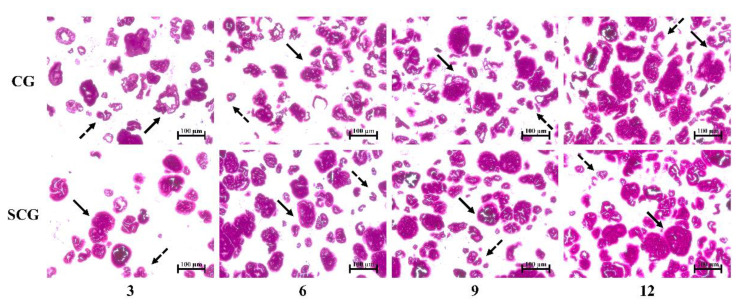
Light microscopy of cooking gels and setting-cooking gels at four starch contents (3%, 6%, 9%, and 12%). 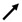 indicates large granule starch; 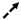 indicates small granule starch. Caption: see Figure 1.

The morphology of potato starch differed appreciably between direct heated gels and two-step heated gels (Figure 3). It was observed that a nearly spherical morphology of starch was shown in SCG, whereas a relatively irregular state was observed in CG. Starch showed non-obstructive expansion in CG with 3% or 6% starch content. The striation structures inside of starch granules were intact with an increase in starch content, showing that the swelling degree of starch decreased in CG. In comparison, it was observed that potato starch granules in SCG were almost circular, especially in the low starch-containing matrices. In addition, large granule starch could not realize valuable swelling in two-step heating. However, regardless of non-setting or setting samples, small starch granules showed extended states, which was more conducive for applying starch in the surimi matrix.

#### 3.2.2. Scanning Electron Micrograph

**Figure 4:**
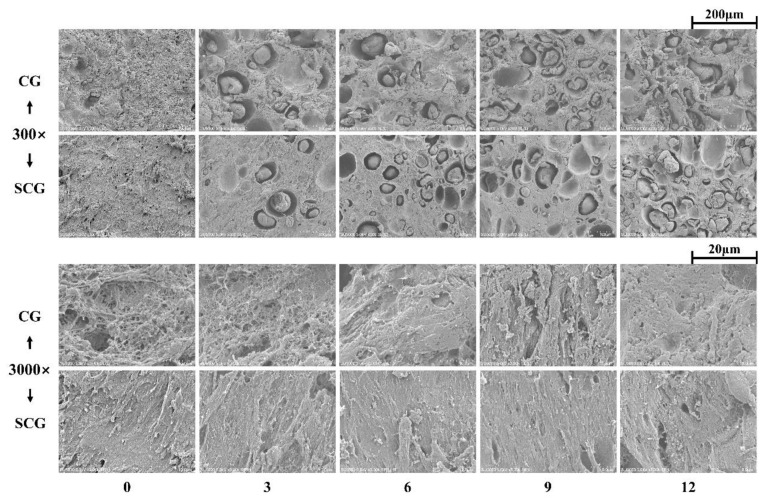
Microstructure micrographs (magnification ×300, ×3000) of starch–surimi matrix subjected with different heating processes: 0, 3, 6, 9, and 12 represented 0%, 3%, 6%, 9%, and 12% starch content, respectively. Caption: See Figure 1.

Overall microstructures of starch–surimi gels were observed at 300× to reflect the morphology of starch and surimi matrix after cooking (Figure 4). The gelatinization temperature of potato starch ranged from 56 to 66 °C, and the amorphous region extended after reaching this temperature range [30]. For CG, as the temperature rose above 45–50 °C, the suwari was partially disrupted, resulting in modori formation and unhindered starch swelling [31]. Moreover, the loose gel network might be a key factor of the irregular swelling of starch. In contrast with non-setting gels, SCG showed more substantial resistance to starch swelling that avoided water loss in the gel network structure. It might contribute to the setting step, which resulted in the formation of a stable surimi gel network in SCG. Thus, it could be determined that the setting effect promoted cross-linking in surimi, hindered water absorption, and broke hydrogen bonds in the striations of starch granules.

Viewed at large magnifications, non-starch containing CG exhibited a coarse matrix with large cavities, as shown in Figure 4. However, the addition of the starch made cavities disappear, resulting in more compact surimi. According to the results from Kong et al. [16], these shrunk surimi networks were considered to be responsible for the “packing effect.” Meanwhile, for SCG, no significant changes were observed, and smooth structures were shown throughout the addition of starch.

### 3.3. Changes in Chemical Interactions of Starch–Surimi Gels with Non-Setting or Setting Effect

#### 3.3.1. Non-Covalent Bonds

Non-covalent bonds play essential roles in supporting the three-dimensional structure and enhancing gel strength [32]. All of the non-covalent bonds (including the non-specific associations, ionic bonds, hydrogen bonds, and hydrophobic interactions) differed between the two types of heating samples (Table 2). The non-specific associations had significant increases in both CG and SCG (*p* < 0.05) with the increase in starch content, which resulted from a weak link with low molecular proteins in the gel [33]. Li et al. [28] discovered that the non-specific associations of myofibrillar protein were affected by starch with a larger particle size. Hence, the increase in non-specific associations might be attributed to the expansion of potato starch. The ionic bonds generated by electrostatic interactions between peptides also increased [34]. Hydrogen bonds could enhance the rigidity of the gel, but it might be easily destroyed under high temperature. However, the reduction in hydrogen bonds, in turn, allowed the hydration of exposed peptide backbones and, thus, was important in stabilizing bound water [2]. Overall, these weak forces uniformly increased with the increment of starch content, containing non-specific associations, ionic bonds, and hydrogen bonds (*p* < 0.05).

Hydrophobic interactions predominate in the gel matrix compared with other non-covalent bonds [17], which are produced by the unfolding action of the protein (above 60 °C) and the exposure of the hydrophobic core. With an increase in starch content, hydrophobic interactions declined in CG (Table 2). The unfolding action of the surimi protein structure during heating contributed to the formation of hydrophobic interactions. It could be presumed that the hydrophilic groups absorbed water, resulting in starch swelling as the temperature rose. Subjected to setting treatment, the hydrophobic interactions tended to increase with relatively minor fluctuations. Compared with CG, the effect of starch addition on hydrophobic interactions in SCG weakened. This was related to the elastic SCG containing disulfide bonds, which had a high extrusion resistance to starch [35].

#### 3.3.2. Total Sulfhydryl Groups

Sulfhydryl groups buried in protein are exposed during heating process and subsequently generate disulfide cross-linking [23]. The disulfide bonds display a type of rheological behavior known as rubber elasticity, and they are critical to maintaining network stability [34]. The concentration of total sulfhydryl groups decreased ceaselessly in CG with increased starch content, as shown in Table 2. It was ascribed to internal changes of protein aggregates in which more peptide chains were unfolded and sulfhydryl groups were exposed. Subsequently, the cross-linking of -SH occurred followed by the formation of more disulfide bonds [36]. Nevertheless, faced with stress from starch, SCG showed minor changes in total sulfhydryl groups, contributing to a more stable structure formed by low-temperature preincubation [37].

### 3.4. FT-IR Spectroscopy Analysis of Starch–Surimi Gels with Non-Setting or Setting Effect

#### 3.4.1. Amide Bands of Protein

**Figure 5:**
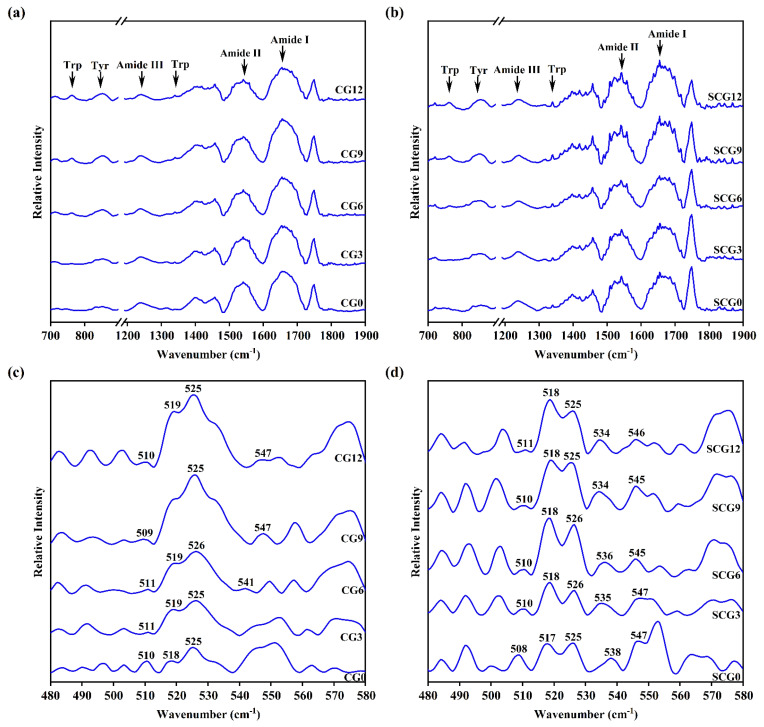
FT-IR spectroscopy (**a**,**b**) and sulfhydryl and disulfides (**c**,**d**) regions of starch–surimi matrix with different treatment. CG0–CG12: cooking gels with starch content (0–12%); SCG0–SCG12: setting-cooking gels with starch content (0–12%).

The gel matrix can be analyzed by using FT-IR spectroscopy to detect functional groups associated with intramolecular and intermolecular structures [38]. The amide bands of proteins have several distinct vibrational modes, including amide I, II, and III. Amide I (1600–1700 cm^−1^) resulted primarily from ν (C=O) and δ(N–H), whereas amide II and III (1480–1580 cm^−1^; 1200–1350 cm^−1^) originated from ν(C–N) and δ(N–H) [39]. Among them, amide I was the most useful in reflecting secondary and tertiary structures [9]. Generally, the α-helix, random coil, β-sheet, and β-turn structures correspond to 1650–1660 cm^−1^, 1660–1665 cm^−1^, 1665–1680 cm^−1^, and –1680 cm^−1^ ranges of the amide I band, respectively [40]. Li et al. [25] discovered that the starch did not cause significant shifts in amide bands (Figure 5a, b, Table 3). Non-setting gels and setting gels both showed peak values of amide I at 1654 cm^−1^, suggesting that α-helix dominated the secondary structure of the protein in the starch–surimi matrix. Although starch could increase the density of the gel matrix and influence chemical interactions, it had little effect on the three-dimensional structure of proteins. In contrast, slight changes in α-helix and random coil occurred between CG and SCG, which was conducive to increasing hydrogen bonds. The α-helix of native and partially denatured proteins and β structures that formed during heating and cooling are both stabilized by hydrogen bonds [2]. Therefore, the secondary structure of surimi protein had no significant change caused by the external physical forces of starch.

#### 3.4.2. Tryptophan (Trp) Residue Bands and Tyrosine (Tyr) Doublet Bands

The vibrations near 760 and 1340 cm^−1^ present the microenvironment of Trp residues. Once the Trp residues buried in the hydrophobic environment were exposed to a polar environment, the intensity of bands showed upward trends [41]. The intensity of tryptophan residue bands increased slightly with the increment of starch content in Figure 4 possibly contributing to the exposure of Trp residues [42]. The starch granules occupied the matrix space, which promoted the unfolding of protein structure and then provided impetus to the exposure of the hydrophobic core. A similar result was found in the Tyr doublet bands, which were proposed as a means for determining whether the tyrosine residue was solvent-exposed or buried [43]. If the intensity at 850 cm^−1^ (I_850_) was higher than I_830_, this indicated that the Tyr residues at this time changed from the “buried” to the “exposed” state [44]. The distinct vibrations were exhibited differently at 830 and 850 cm^−1^ in both gels (Figure 5a, b). A decreased I_830_ and a concurrent increased I_850_ indicated that there were weak hydrophobic interactions among tyrosine residues. It could be demonstrated that protein solubility of CG increased due to reduced hydrophobic interactions. However, the change of hydrophobic amino acids between CG and SCG was not obvious. Therefore, the setting treatment of surimi did not hinder the effect of starch addition on the spatial structure of hydrophobic amino acids in surimi proteins.

#### 3.4.3. S-S Stretching

The formation and interchange reactions of disulfide are important for non-reversible heat gelation [9]. Moreover, spectral features in the 500–550 cm^−1^ region were correlated with the structural parameters of the disulfide bands (Figure 5c, d). Stretching vibrations located at 510 cm^−1^, 516–530 cm^−^^1^, and 535–545 cm^−1^ have been assigned to gauche–gauche–gauche, gauche–gauche–trans, and trans–gauche–trans, where the –C–S–S–C– torsion angle of 90° was represented as gauche form and that of 180° was represented as trans form [45]. Due to its low potential energy, the gauche–gauche–gauche form was presumably the most stable among the others [46]. Quantitative analysis of FT-IR spectroscopy showed stable bands located at 510 cm^−1^ in CG and SCG and disappeared with the increment of starch addition. The weak bands vibrations of CG significantly increased at 516–530 cm^−1^. It meant that not only disulfide bond conformation changed from gauche–gauche–gauche to gauche–gauche–trans but also new bonds emerged. The variation was consistent with the decrease in total sulfhydryl groups, indicating that more disulfide bonds formed in CG. Therefore, the conformational shift occurred by a combination with excessive starch, which was conducive to the breaking and reformation of disulfide bonds during the direct heating process. The vibrations rose near 518 cm^−1^ and 525 cm^−1^ in SCG, accompanied by a decrease in bonds near 510 cm^−1^. Thus, the setting effect resulted in micro-changes in the conformation of the disulfide bonds in SCG. In brief, the results showed that the matrix after the setting process had a specific compressive capacity, causing fewer vibrations (S–S) changes.

### 3.5. Changes in Gel Properties of Starch–Surimi Gels with Non-Setting or Setting Effect

#### 3.5.1. Texture Profile Analysis

Texture profile analysis simulates chewing twice to obtain the characteristic parameters of the gel matrix (Table 4) [47]. A significant increase in hardness was observed along with the elevated starch content due to reduced moisture content and starch swelling. The starch-absorbed water and swelled during the thermal processing, which increased pressure on the surimi gel [22]. No significant difference in springiness occurred in CG with the addition of starch (*p* > 0.05). However, springiness decreased along with the addition of starch in SCG. It is speculated that if the surimi gel had better gelling properties, then it would play a dominant role in the mixed matrix. Thus, the reduction in springiness in SCG was attributed to decreased surimi concentration. The improvement of cohesiveness and resilience in CG presumably contributed to granule-swelling of starch. The gelation and swelling of starch granules might cause an increase in hydration, which enabled better compatibility of protein networks and starch [14]. SCG showed reduced cohesiveness and resilience with excessive starch, which could be explained by how excessive expansion in starch granules attenuated the stability of the surimi matrix. Thus, potato starch filling could ameliorate the weak gel matrices by increasing texture properties such as hardness and cohesiveness. Nevertheless, the addition of potato starch increased the hardness of the gels but at the same time reduced springiness and cohesiveness. However, compared with a large content of starch, CG still showed lower textural properties, which was connected with the limited filling capacity of natural potato starch and the poor texture of unsetting surimi gel.

#### 3.5.2. Whiteness

Whiteness is one of the most essential indications of surimi quality. The variation in whiteness is found to be correlated not only to the conformation of the gel network but also to the color of additives [48,49]. As presented in Table 5, significant increases in whiteness were found with starch contained in both two types of gels (*p* < 0.05). Moreover, two-step heated gels showed higher whiteness in different starch content compared with direct heating gels. The differences in two types of gels might be attributed to a restrictive effect on starch swelling. L*, a*, and b* values decreased as potato starch content increased and presented significant differences between CG and SCG.

#### 3.5.3. Water Holding Capacity

**Figure 6:**
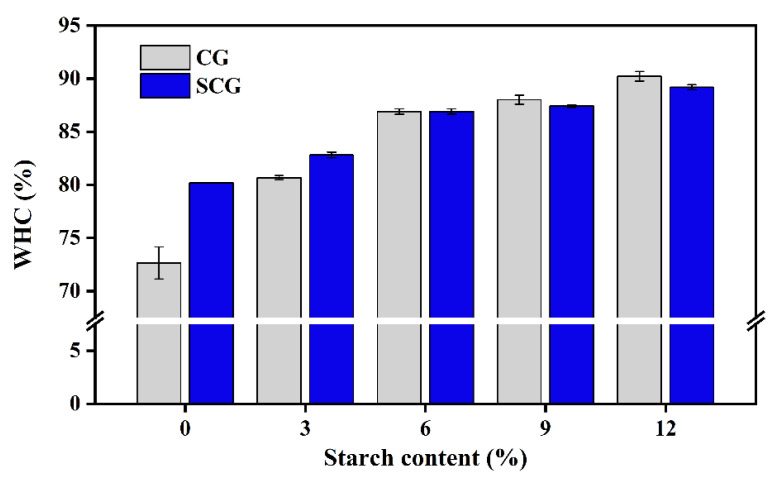
Water holding capacity of different water in starch–surimi matrix subjected to direct heating and two-step heating. Caption: see Figure 1.

WHC represents the stability of the mixed matrix suffering external forces, which indirectly reflects the compactness of surimi gels and water absorption capacity of starch [50]. As shown in Figure 6, the gel matrix presented a significant increase in WHC accompanied by adding starch in both heating processes (*p* < 0.05). This was in line with previous research by Mi et al. [17] and Luo et al. [14]. It has been revealed that starch had strong hydrophilicity and showed an effective swelling effect with larger particles, which increases the WHC of the gel matrix. The setting effect promoted the formation of the myofibrillar protein network, which could also reduce water loss caused by an external force, thereby increasing WHC. The matrix treated by direct heating showed better WHC with a continuous increase in starch content. It was related to the irregular expansion of starch confirmed by the microstructure (Figure 4). In addition, WHC was markedly correlated with the variations in peak area proportion and peak relaxation time [51]. Some possible assumptions could be introduced to describe the changes of WHC: (i) the increase in WHC was associated with the migration of free water by adding starch; (ii) starch showed better swelling states in CG on account of the higher T_P2b_ and lower T_P22_; and (iii) the setting effect improved the stability of immobile water, whereas it limited starch swelling.

## 4. Conclusions

Changes in the water migration and distribution, tissue microstructures, chemical interactions, protein spatial structure, and physical properties were studied. The addition of starch expanded and caused moisture migration, which was ascribed to the combination of free water and hydrophilic groups in starch. The competition between surimi and starch for water affected the chemical interactions and forces in surimi gel components, thereby indirectly resulting in changes in the microstructure and water holding properties of the gel matrix. For the direct cooking matrix, the porous surimi gel lost water when it was squeezed by starch, resulting in a reduction in pore size and immobile water inside. Moreover, starch was not conducive to the interactions of hydrophobic groups, whereas weak disulfide bonds were generated after adding starch. The enhancement of textural properties might be related to changes in protein structure. Subjected to the setting process, the matrix showed an ascendant trend in non-specific associations, ionic bonds, and hydrogen bonds, which was similar to non-setting gels. Setting-cooking gels with a compact structure were less affected by starch-swelling. However, adding excessive starch destroyed the integrity of SCG and decreased cohesiveness and resilience. Therefore, this study found that the addition of starch content had a better supporting effect on weak gels, which provided some insights for improving the quality of surimi-based products. Moreover, further studies are needed to improve the compatibility and active filling of starch in the two-step heating process.

## Figures and Tables

**Table 1:** CPMG parameters.

CPMG	SF	SW	RFD	Tw	RG1	DRG1	PRG	P1	P2	TE	NECH	NS
Parameter	21	200	0.08	2500	10	3	1	20	40	0.3	8000	8

SF: magnet frequency, MHz; SW: spectral width, kHz; RFD: radio frequency delay, ms; Tw: recycle delay, ms; RG1: regulate analog gain 1, db; DRG1: regulate digital gain 1; PRG: pre-amplified receiver gain; P1: 90° pulse lengths, μs; P2: 180° pulse lengths, μs; TE: pulse gaps between π and π, ms; NECH: echo number; NS: scanning number.

**Table 2:** Non-covalent bonds and total sulfhydryl groups of starch–surimi mixtures subjected with different heating processes.

Samples	Starch Content (%)	Non-Covalent Bonds (g/L)				Total Sulfhydryl Groups (μmol/g Protein)
		Non-Specific Associations	Ionic Bonds	Hydrogen Bonds	Hydrophobic Interactions	
CG	0	0.99 ± 0.03 ^Be^	0.85 ± 0.02 ^Bd^	0.39 ± 0.02 ^Bd^	7.71 ± 0.35 ^Aa^	25.17 ± 0.96 ^Aa^
	3	1.22 ± 0.01 ^Bd^	1.33 ± 0.02 ^Ac^	0.63 ± 0.03 ^Bc^	7.75 ± 0.27 ^Aa^	20.07 ± 1.75 ^Ab^
	6	1.30 ± 0.02 ^Bc^	1.90 ± 0.03 ^Ab^	1.21 ± 0.01 ^Bb^	5.64 ± 0.10 ^Ab^	19.44 ± 2.60 ^Ab^
	9	2.00 ± 0.01 ^Bb^	1.84 ± 0.03 ^Ab^	2.19 ± 0.04 ^Aa^	4.84 ± 0.10 ^Ac^	18.29 ± 1.34 ^Bb^
	12	2.60 ± 0.06 ^Ba^	2.16 ± 0.06 ^Aa^	2.21 ± 0.02 ^Ba^	3.94 ± 0.10 ^Ad^	12.55 ± 1.10 ^Bc^
SCG	0	1.58 ± 0.05 ^Ae^	1.03 ± 0.03 ^Ad^	1.16 ± 0.02 ^Ae^	2.95 ± 0.09 ^Be^	25.62 ± 1.91 ^Aa^
	3	1.97 ± 0.03 ^Ad^	1.21 ± 0.02 ^Bc^	1.82 ± 0.01 ^Ad^	4.06 ± 0.04 ^Ba^	21.03 ± 2.02 ^Abc^
	6	2.43 ± 0.02 ^Ac^	1.25 ± 0.03 ^Bc^	2.69 ± 0.04 ^Aa^	3.47 ± 0.02 ^Bb^	17.97 ± 1.75 ^Ac^
	9	2.96 ± 0.04 ^Ab^	1.33 ± 0.02 ^Bb^	2.19 ± 0.01 ^Ac^	3.41 ± 0.03 ^Bc^	23.20 ± 3.09 ^Aab^
	12	4.07 ± 0.05 ^Aa^	1.56 ± 0.01 ^Ba^	2.36 ± 0.03 ^Ab^	3.17 ± 0.03 ^Bd^	22.43 ± 1.89 ^Aab^

Uppercase letters indicate significant difference (*p* < 0.05) between different heating processes, lowercase letters indicate the difference between gels with different starch content (*p* < 0.05), and the values are expressed as mean ± SD.

**Table 3:** Secondary structure (amide I) of protein in different starch–surimi matrices.

Samples	Starch Content (%)	α-Helix (%)	Random Coil (%)	β-Sheet (%)	β-Turn (%)
CG	0	26.16 ± 0.75 ^Aa^	26.49 ± 0.26 ^Aa^	25.49 ± 0.18 ^Aa^	21.85 ± 0.73 ^Aa^
	3	26.89 ± 1.38 ^Ba^	25.80 ± 1.13 ^Aa^	25.20 ± 0.96 ^Aa^	22.11 ± 2.84 ^Aa^
	6	26.47 ± 1.07 ^Aa^	26.03 ± 0.53 ^Aa^	25.63 ± 0.61 ^Aa^	21.88 ± 1.02 ^Aa^
	9	27.20 ± 1.01 ^Ba^	26.00 ± 0.23 ^Aa^	25.05 ± 0.47 ^Aa^	21.75 ± 0.35 ^Aa^
	12	27.69 ± 1.03 ^Ba^	25.97 ± 0.21 ^Aa^	25.34 ± 0.16 ^Aa^	21.00 ± 1.06 ^Aa^
SCG	0	28.14 ± 2.39 ^Aa^	25.03 ± 0.75 ^Ba^	24.47 ± 1.32 ^Ab^	22.36 ± 2.34 ^Aa^
	3	28.98 ± 0.55 ^Aa^	25.25 ± 0.32 ^Aa^	24.92 ± 0.29 ^Aab^	20.85 ± 0.26 ^Aa^
	6	27.74 ± 1.19 ^Aa^	24.82 ± 0.31 ^Ba^	25.89 ± 0.15 ^Aa^	21.55 ± 0.99 ^Aa^
	9	29.12 ± 0.62 ^Aa^	24.66 ± 0.48 ^Ba^	25.65 ± 0.47 ^Aa^	20.56 ± 0.49 ^Ba^
	12	29.03 ± 0.38 ^Aa^	25.17 ± 0.40 ^Ba^	25.13 ± 0.31 ^Aab^	20.67 ± 0.52 ^Aa^

Uppercase letters indicate significant difference (*p* < 0.05) between different heating processes, lowercase letters indicate the difference between gels with different starch content (*p* < 0.05), and values are expressed as mean ± SD.

**Table 4:** Textural properties of starch–surimi mixtures subjected with different heating processes.

Samples	Starch Content (%)	Hardness (g)	Springiness	Cohesiveness	Resilience	Chewiness (g)
CG	0	476.41 ± 8.31 ^Be^	0.94 ± 0.00 ^Ba^	0.73 ± 0.00 ^Be^	0.40 ± 0.00 ^Be^	325.83 ± 4.29 ^Be^
	3	757.80 ± 20.71 ^Bd^	0.95 ± 0.01 ^Aa^	0.73 ± 0.00 ^Bd^	0.42 ± 0.00 ^Bd^	528.43 ± 20.04 ^Bd^
	6	1097.31 ± 11.53 ^Bc^	0.96 ± 0.01 ^Aa^	0.74 ± 0.00 ^Bc^	0.43 ± 0.00 ^Bc^	780.30 ± 2.10 ^Bc^
	9	1382.00 ± 17.42 ^Bb^	0.94 ± 0.02 ^Aa^	0.75 ± 0.00 ^Bd^	0.44 ± 0.00 ^Bb^	983.86 ± 13.48 ^Bb^
	12	1559.28 ± 17.46 ^Ba^	0.91 ± 0.01 ^Ab^	0.76 ± 0.00 ^Ba^	0.45 ± 0.00 ^Ba^	1090.18 ± 20.44 ^Ba^
SCG	0	868.25 ± 20.61 ^Ae^	0.97 ± 0.00 ^Aa^	0.85 ± 0.01 ^Aa^	0.59 ± 0.00 ^Aa^	716.50 ± 18.45 ^Ae^
	3	1023.91 ± 17.23 ^Ad^	0.96 ± 0.02 ^Aab^	0.84 ± 0.00 ^Ab^	0.57 ± 0.00 ^Ab^	826.50 ± 30.43 ^Ad^
	6	1301.55 ± 27.10 ^Ac^	0.94 ± 0.00 ^Abc^	0.82 ± 0.00 ^Ac^	0.54 ± 0.01 ^Ac^	1005.24 ± 20.65 ^Ac^
	9	1595.79 ± 4.69 ^Ab^	0.93 ± 0.01 ^Ac^	0.81 ± 0.00 ^Ad^	0.52 ± 0.00 ^Ad^	1200.29 ± 5.83 ^Ab^
	12	1874.12 ± 26.36 ^Aa^	0.92 ± 0.01 ^Ac^	0.80 ± 0.00 ^Ae^	0.51 ± 0.00 ^Ae^	1383.62 ± 26.91 ^Aa^

Uppercase letters indicate significant difference (*p* < 0.05) between different heating processes, lowercase letters indicate the difference between gels with different starch content (*p* < 0.05), and values are expressed as mean ± SD.

**Table 5:** Whiteness of starch–surimi mixtures subjected with different heating processes.

Samples	Starch Content (%)	L *	a *	b *	Whiteness
CG	0	77.45 ± 0.17 ^Aa^	−0.54 ± 0.12 ^Aa^	6.06 ± 0.35 ^Aa^	76.64 ± 0.14 ^Ba^
	3	75.51 ± 0.31 ^Ab^	−0.60 ± 0.08 ^Aa^	5.77 ± 0.15 ^Ab^	74.83 ± 0.28 ^Bb^
	6	73.95 ± 0.39 ^Bc^	−0.73 ± 0.05 ^Ab^	5.63 ± 0.17 ^Ab^	73.33 ± 0.36 ^Bc^
	9	71.65 ± 0.17 ^Bd^	−1.02 ± 0.08 ^Ac^	4.96 ± 0.18 ^Ac^	71.20 ± 0.19 ^Bd^
	12	71.06 ± 0.28 ^Be^	−1.06 ± 0.05 ^Ac^	4.67 ± 0.06 ^Ad^	70.67 ± 0.27 ^Be^
SCG	0	77.46 ± 0.24 ^Aa^	−0.83 ± 0.06 ^Ba^	5.19 ± 0.16 ^Ba^	76.86 ± 0.25 ^Aa^
	3	75.77 ± 0.35 ^Ab^	−0.88 ± 0.04 ^Bb^	4.92 ± 0.22 ^Bb^	75.26 ± 0.31 ^Ab^
	6	74.87 ± 0.33 ^Ac^	−0.97 ± 0.05 ^Bc^	4.79 ± 0.14 ^Bb^	74.40 ± 0.34 ^Ac^
	9	73.32 ± 0.24 ^Ad^	−1.07 ± 0.05 ^Bd^	4.46 ± 0.20 ^Bc^	72.93 ± 0.21 ^Ad^
	12	71.84 ± 0.21 ^Ae^	−1.28 ± 0.04 ^Be^	4.07 ± 0.06 ^Bd^	71.51 ± 0.21 ^Ae^

Uppercase letters indicate significant difference (*p* < 0.05) between different heating processes, lowercase letters indicate the difference between gels with different starch content (*p* < 0.05), and values are expressed as mean ± SD. L *: lightness; a *: red-green value; b *: yellow-bule value.

## Data Availability

Not applicable.
